# Pathophysiology of GvHD and Other HSCT-Related Major Complications

**DOI:** 10.3389/fimmu.2017.00079

**Published:** 2017-03-20

**Authors:** Sakhila Ghimire, Daniela Weber, Emily Mavin, Xiao nong Wang, Anne Mary Dickinson, Ernst Holler

**Affiliations:** ^1^Department of Internal Medicine III, University Medical Centre, Regensburg, Germany; ^2^Hematological Sciences, Institute of Cellular Medicine, Newcastle University, Newcastle, UK

**Keywords:** pathophysiology, graft versus host disease, T cells, haematopoietic stem cell transplantation, prophylaxis

## Abstract

For over 60 years, hematopoietic stem cell transplantation has been the major curative therapy for several hematological and genetic disorders, but its efficacy is limited by the secondary disease called graft versus host disease (GvHD). Huge advances have been made in successful transplantation in order to improve patient quality of life, and yet, complete success is hard to achieve. This review assimilates recent updates on pathophysiology of GvHD, prophylaxis and treatment of GvHD-related complications, and advances in the potential treatment of GvHD.

## Introduction to Graft Versus Host Disease (GvHD)

Graft versus host disease is the most recognized complication post-hematopoietic stem cell transplantation (HSCT) and was first observed in 1956 in a murine model. Barnes and Loutit demonstrated that when irradiated mice were infused with allogenic marrow and spleen cells, mice recovered from radiation injury and aplasia but they developed diarrhea, weight loss, skin changes, and liver abnormalities, and subsequently died due to “secondary disease” (1). This phenomenon was recognized as GvHD. A decade later, in 1966, Billingham postulated three crucial requirements for the development of GvHD:

(i) the transplanted graft must contain immunologically competent cells,
(ii) the recipient must be incapable of rejecting or eliminating transplanted cells,
(iii) the recipient must express tissue antigens that are not present in the transplant donor, thus the recipient antigens are recognized as foreign by donor cells (2).

Today, we know that the immunocompetent cells are T lymphocytes that are present in the stem cell inoculum and are required to mount an effective immune response (3). A normal immune system is able to reject T cells from a foreign donor. However, when recipient’s immune system is compromised through the use of various immune-ablative agents (chemotherapy and/or radiotherapy), the recipient is incapable of rejecting the transplanted cells. We now know that the tissue antigens that differ in donor and recipient are major and minor human leukocyte antigens (HLA), and their expression on cell surfaces is crucial for the activation of allogenic T cells and initiation of GvHD (4). Previously, it was believed that acute GvHD occurs within day 100 after transplantation and chronic GvHD (cGvHD) occurs beyond day 100 and that the most affected organs at the onset of GvHD are skin (81%), gastrointestinal tract (54%), and liver (50%) (4). Now, it is clear that acute GvHD can occur after day 100 as late acute GvHD (e.g., after cessation of immunosuppression or after donor lymphocyte infusion) or cause overlap syndrome of both acute GvHD and cGvHD (5).

## Pathophysiology of Acute GvHD: A Three-Step Model Explaining the Current Strategies of Prophylaxis and Treatment

Acute GvHD has been attributed to three stages. Initially, there is tissue damage due to conditioning that in turn activates the host antigen-presenting cells (APCs). Secondly, APCs activate donor T cells, also known as an afferent phase. Finally, in efferent phase, cellular and inflammatory factors work together to damage the target organs.

### Conditioning-Mediated Tissue Damage

Conditioning is crucial to eradicate underlying disease and to support engraftment of donor cells without rejection by recipient (6). Prior to donor cell infusion, patient’s tissues have been profoundly damaged due to underlying disease itself, treatment for the disease, infections, and the conditioning regimen (7, 8). As a consequence, damaged host tissue releases danger signals, which include pro-inflammatory cytokines such as tumor necrosis factor (TNF) and interleukin-1 (IL-1) (9), that activate host APCs, ultimately activating donor T cells present in the stem cell inoculum (10, 11). Conditioning-mediated damage to the gastrointestinal (GI) tract remains the main concern as GI tract allows systemic translocation of microbial products like lipopolysaccharide (LPS) and other pathogen associated molecular patterns that greatly amplify host APC activation (8), leading to amplified T-cell activation. Conditioning-related damage also explains why the concept of reduced intensity or even non-myeloablative conditioning has contributed to less toxicity, less severe GvHD, and reduced treatment-related mortality. Some studies showed that delaying the transfer of donor cells after conditioning decreased the risk of GvHD (9, 12).

### Donor T Cell Activation (the Afferent Phase)

Graft versus host disease occurs when donor T cells activate and respond to HLA differences on recipient’s tissue (13). Experimental models have proved that the host APCs are necessary and sufficient to activate donor T cells and initiate GvHD (11, 14). Donor T cells can recognize alloantigen either on host APC, known as direct antigen presentation (15), or on donor APCs, known as indirect presentation (16). T-cell responses depend on the disparity between the donor and the recipient with regard to HLA (13). CD4+ T cells respond to the variations in MHC class II molecule (HLA-DR, -DQ, and -DP), and CD8+ T cells respond to the variations in MHC class I molecule (HLA-A, -B, and -C) (17). Transplants carried out in the HLA-matched sibling or identical twin setting can still give rise to GvHD due to differences in minor HLA (18). The first to be described were HA-1 (19) and HA2 (20), and the subsequent clinical impact of minor histocompatibility antigens including H-Y antigens (21, 22) of female-to-male transplants has recently been reviewed (23, 24). Minor HLAs are T-cell epitopes, which are originally derived from polymorphic or normal tissue proteins. These antigenic peptides can be presented on HLA Class I or Class II molecules, and to date over 50 minor HLA antigens have been identified (24). Minor HLA antigens have been associated with GvHD and graft versus leukemia (GvL) effects due to their tissue distribution. Minor HLA antigens restricted to the hematopoietic system may be able to enhance GvL responses while more broadly expressed minor HLA antigens contribute to both GvHD and GvL (25). As well as cytotoxic T-cell responses of allogeneic H-Y antibodies have shown to predict cGvHD and non-relapse mortality (26, 27).

T-cell activation is in the focus of current immunosuppressive strategies used for prophylaxis and treatment. Calcineurin inhibitors, mycophenolate, and mToR inhibitors interfere with different signals of T-cell activation (28, 29). The broader strategy is T-cell depletion, which is currently applied by *in vivo* approaches such as the use of antithymocyte globulin pretransplant (30). Cytotoxic approaches more or less selectively eliminate activated T cells if applied posttransplant; the old approach of methotrexate prophylaxis but also the more recent approach of using posttransplant cyclophosphamide engages this principle (31).

### Target Cell Apoptosis (the Efferent Phase)

In this phase, both innate and adaptive immune cells work synergistically to exacerbate the T cell-induced inflammation. Cellular mediators, such as cytotoxic T lymphocytes (CTLs) and natural killer (NK) cells, utilize the Fas/Fas ligand (FasL) pathway and perforin/granzyme pathway to lyse the target cells (32, 33). Furthermore, inflammatory cytokines synergize with CTLs, resulting in further tissue injury and possible target organ dysfunction (13). In addition, microbial products like LPS, released during conditioning, leak through a damaged intestinal mucosa and skin and stimulate mononuclear cells (monocytes/macrophages) to secret inflammatory cytokines leading to amplification and propagation of a cytokine storm (13). This leads to destruction of epithelial cells, mostly in the GI tract.

The broad activity of corticosteroids including induction of T-cell apoptosis, suppression of macrophage activation, and cytokine release explains why these old drugs are still the treatment of choice for first-line treatment of both acute GvHD and cGvHD. Cytokine inhibitors like TNF blocking agents were thought to be more specific but did not result in increased response rates (34). For almost all second-line strategies in steroid-refractory acute GvHD low response rates associated with high treatment-related mortality have been reported that the urgent need for further improvement (35).

In the last 10 years, the concept of GvHD pathophysiology has been largely extended, and a more differentiated view has been adapted.

Firstly, the mechanism of conditioning-related damage has further been specified. It is now clear that the tissue damage results in release of several danger signals such as uric acid and the metabolites of adenosine triphosphate pathway and its receptor has been shown to be involved in activation of GvHD (36).

Secondly, the concept of LPS-triggered inflammation has been substituted by multiple microbiota derived signals and differential activation of toll-like receptors (TLRs) and NOD-like receptors (NLRs). NOD2/CARD15 has been shown to be involved in triggering the inflammation both in mice (37, 38) and men (39). More recently, it became clear that the microbiota of epithelial tissues is the major player influencing epithelial integrity and local immune tolerance by commensal bacteria and millions of metabolites are produced to maintain epithelial homeostasis (40, 41).

**Figure 1:**
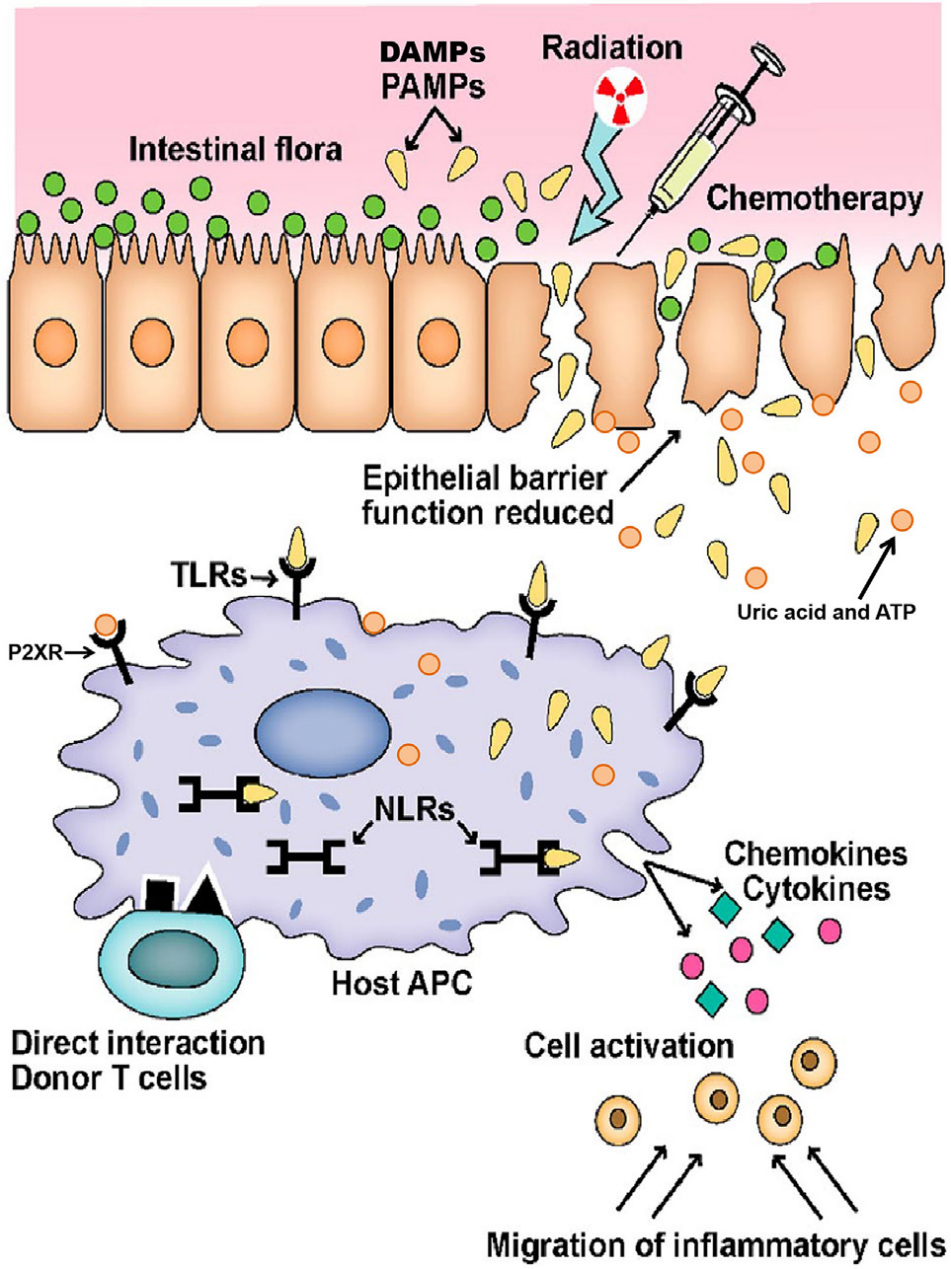
**Initiation of graft versus host disease**. Conditioning regimen leads to destruction of epithelial cells and their integrity. Damaged epithelia secrete uric acid and adenosine triphosphate (ATP) that result in production of pro-inflammatory cytokines. Pathogen recognition receptors, such as toll-like receptors (TLRs), NOD-like receptors (NLRs), and P2XRs, are activated by pathogen associated molecular patterns (PAMPs) and danger associated molecular patterns (DAMPs). These signals ultimately activate antigen-presenting cells (APCs) that lead to donor T-cell activation. Adopted and modified from Ref. (37).

**Figure 2:**
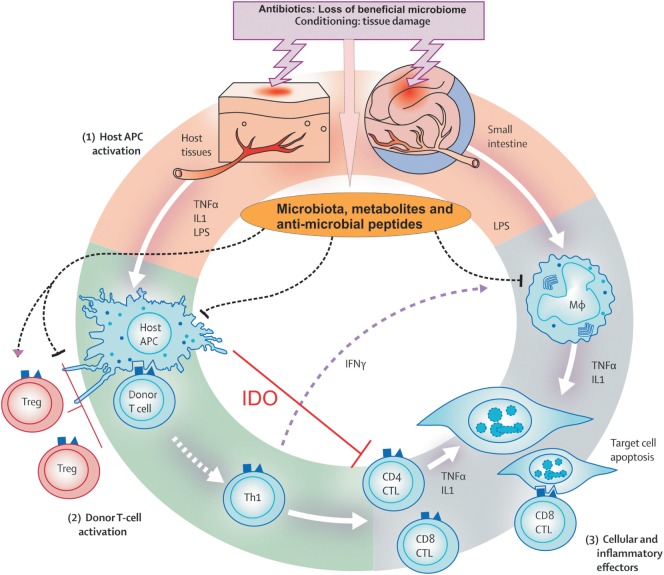
**Pathophysiology of acute graft versus host disease**. Conditioning regimen cause profound damage to the host tissues leading to release of inflammatory cytokines like tumor necrosis factor and interleukin-1. These cytokines activate host antigen-presenting cells (APCs) in phase I. In addition, loss of microbial diversity and metabolites thereof leads to loss of epithelial and immune homeostasis. Host APCs activate mature donor T cells present in stem cell inoculum in phase II. T cells subsequently proliferate and differentiate into Th1 and Th17 type, which are involved in activation of CD4 cytotoxic T lymphocyte (CTL), CD8 CTL, and natural killer cells that mediate tissue damage. In phase III, effector T cells together with pro-inflammatory cytokines attack the epithelial cells of skin, liver, lung, and gastrointestinal tract. This damage is further supported by the lipopolysaccharide (LPS) that has leaked through damaged intestinal mucosa, which then recruits myeloid cells to further produce pro-inflammatory cytokines and thus enhance the cytokine storm. Adopted and modified from Ref. (13).

Finally, and in context with the concept of microbiota as important players, the importance of regulatory immune cells that balance immune reactions is recognized. Regulatory T cells (Tregs) expressing the transcription factor Foxp3 occur as natural, thymus derived T cells and are able to prevent alloreaction (42). On epithelial surfaces, induced peripheral Tregs try to dampen acute inflammation (43). Foxp3 positive T cells act in cooperation with numerous newly identified regulatory populations, such as invariant natural killer T cells (44), myeloid-derived suppressor cells (MDSCs), and a whole new set of innate immune cells such as innate lymphoid cells (45). Figure 1 represents the GvHD initiation phase. Figure 2 summarizes the complete pathophysiology of aGvHD.

## *In vitro* Modeling of GvHD to Give Insight into the Pathophysiology

**Figure 3:**
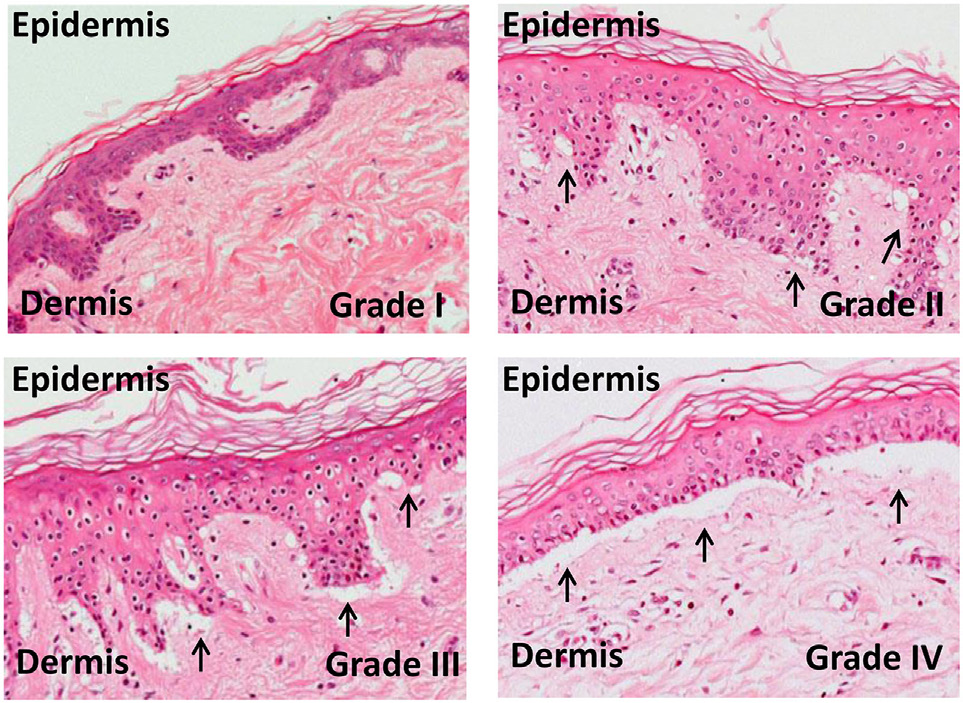
**Skin explant grades I–IV**. The outcome of the skin explant assay is histopathological damage ranging from grade I GvHR (with minimal vacuolization in the epidermis) to grade II GvHR (with vacuolization and dyskeratotic bodies) to grade III GvHR (with sub epidermal cleft formation) and finally to grade IV GvHR (with separation of the dermis from the epidermis).

The skin explant model has long been established as a tool for studying the immunobiology of GvHD (Figure 3) and more recently has been used to investigate the specificity of antiviral T cells in graft versus host (GvH) reactions (34–36), the role of Tregs, and mechanisms of apoptosis (46, 47).

The skin explant model has also been used to assess the safety of *ex vivo* expanded Treg cells as well as their capacity to prevent GvH reactions (48). Activated and expanded polyclonal Treg, at any cell concentration, did not induce any significant GvH reactions.

Over recent years, significant advances in the understanding of the benefits of Tregs in hematopoietic stem cell transplantation have resulted in the completion of early stage clinical trials as well as the initiation of trials in solid organ transplantation (49–52). These early stage HSCT trials have provided promising results showing a reduction in the incidence of GvHD without adversely effecting relapse, transplant-related mortality, and engraftment. Using the skin explant model, it has been possible to investigate the cellular and molecular mechanisms by which Tregs are likely to be preventing GvHD following HSCT.

We have shown that for Treg to suppress GvH reactions they need to be present during the priming of alloreactive T cells (48). Polyclonal Treg cells were expanded *ex vivo* and added into the skin explant model at either the priming or the effector stage. The later addition of Treg, during the effector phase, impaired their suppressive capacity. This suggests that Treg may be more effective when given early, as prophylaxis, rather than as a treatment. This study also demonstrated that in humans an effector to Treg ratio of 4:1 was sufficient to modulate GvH reactions, whereas previous studies in mice had suggested a 1:1 ratio was necessary. This study has therefore provided preclinical evidence to support the safety and feasibility of *ex vivo* expanded Treg as a novel therapeutic and provides information on the optimal timing and dose of Treg to prevent GvH reactions.

Further work using the skin explant model has been able to elucidate some of the mechanisms by which Tregs are able to prevent GvHR. The presence of Treg during the priming of alloreactive T cells reduced their cytotoxic capacity (48). Further investigations showed that Treg also impaired the ability of alloreactive T cells to migrate into the target tissues (53). The presence of Treg during priming resulted in a reduction in IFNγ production by CD8+ cytotoxic T cells, as well as a reducing expression of skin homing molecules CXCR3 and CLA. This paired with a reduction in levels of the chemokines CXCL10 and CXCL11 in the skin resulted in a significant reduction in the number of cytotoxic T cells present in the skin and decreased the GvH severity. We have since demonstrated that Tregs are able to modulate GvH reactions through impairment of dendritic cells at a transcriptional level, arresting them in a semi-mature status and leaving them functionally impaired (54).

The skin explant model has also been used to investigate the involvement of epithelial Fas in the pathophysiology of GvHD (55). Animal models have previously shown the critical role for Fas/FasL in GvHD (56). Ruffin et al. showed that there was a significant increase in Fas expressing cells in GvHR positive experiments and that Fas-mediated apoptosis was involved in the induction of GvHR, as blocking Fas-mediated apoptosis reduced the severity of GvHR. They also showed that levels of Fas in the serum of patients who received myeloablative conditioning were increased, possibly due to the higher toxicity. This supports the potential use of Fas as a therapeutic target.

## Identification of Biomarkers

As well as investigating the safety of cellular therapies and immunology of GvHD, the skin explant model has been used in recent years to identify biomarkers. Within our group, we used the skin explant model to validate a number of biomarkers, which had been identified in the serum of HSCT patients (57). BAFF and IL-33 levels were elevated pretransplant in patients who then went on to develop aGvHD and therefore could have the potential to act as predictive biomarkers. We also found that CXCL10 and CXCL11 were suitable as diagnostic markers of GvHD. Training and validation cohorts were used to highlight the association of these potential biomarkers to GvHD. Then the skin explant model was used to confirm their association with GvH reactions. Immunohistochemistry was carried out on sections from the skin explant, and increased staining for BAFF, IL-33, CXCL10, and CXCL11 was seen in skin explants with a higher grade GVHR. This was further confirmed in clinical biopsies demonstrating increased levels of protein, measured with immunohistochemistry and gene expression for BAFF, CXCL10, and CXCL11. In this study, the skin explant proved to be a useful tool in validating a panel of biomarkers, which had been identified in patient samples. The skin explant is not exclusive to the human setting. Recently, Zinöcker et al. have described the use of a rat skin explant model for investigating the pathophysiology of GvHD (58) as well as gene expression profiling (59).

Harris et al. (60) have recently reviewed the use of biomarkers in predicting acute GvHD, which include genomic factors as well as plasma proteins. One of the first studies demonstrated that a panel of tumor necrosis factor receptor type 1 (TNFR1) interleukin-2 receptor alpha, IL-8, and hepatocyte growth factor (HGF) had prognostic as well as diagnostic value in predicting acute GvHD (61). Other markers in the skin such as elafin (61) and plasma biomarkers of the lower GI tract and liver acute GvHD have been validated in subsequent studies, and the most significant of these was regenerating islet-derived 3 alpha (Reg3a) (62, 63). These studies led to the use of the biomarkers TNFR1, ILRα, IL-8, HGF, Reg3α, and elafin for measuring responsiveness to GvHD therapy. The panel was able to predict 28-day post-therapy non-response and 180-day mortality in a cohort of 112 patients (64).

In addition an algorithm using concentrations of three biomarkers TNFR1, soluble IL-33 receptor (ST2), and Reg3α, Levine and colleagues (65) were able to calculate the probability of non-relapse mortality caused by non-responsive GvHD and divide the patients into distinct groups to predict response to GvHD therapy. The researchers subsequently developed the Mount Sinai Acute GvHD International Consortium, which consists of a group of 10 transplant centers in the United States and Europe who collaborate on the use of this scoring system to test new treatments for acute GvHD.

## Pathophysiology of cGvHD

Although the pathophysiology of cGvHD is poorly understood, it remains the major cause of late non-relapse death after HSCT (66). cGvHD may manifest simultaneously from aGvHD, develop after the treatment of aGvHD, or may occur *de novo* (67). Classical cGvHD occurs 100 days after transplantation but may also overlap with aGvHD (5, 68).

Acute GvHD is a major risk factor of cGvHD and strategies aiming at T-cell depletion at the time of transplantation to prevent cGvHD demonstrate that early events impact on the development of cGvHD. As immune cells and immune organs, such as thymus, bone marrow niche, and spleen, are the primary targets of acute GvHD, thymus destruction and deficient selection of donor T cells by the thymus are the major factors resulting in allo- and autoimmunity associated with cGvHD (69). Due to early damage of the B cell niche in the bone marrow, B cell development is strongly disturbed resulting in elevated BAFF levels as a predictor of cGvHD and insufficient elimination in B cells producing auto- and alloantibodies (70). A hallmark of cGvHD is development of sclerotic lesions, which can occur in almost every organ (68). While previous data favor a concept of defective wound healing with increase production of sclerotic cytokines, such as TGFβ and PDGF, recent evidence supports a role of specific TH17 subsets in this sclerotic process (71).

## Target Organ Damage During GvHD

Skin is the principal target organ of GvHD, and the initial manifestation in the skin is maculopapular rash, which has the potential to spread throughout the body (13). The rash may resemble folliculitis or may resemble sunburn. In extreme cases, skin may blister and ulcerate (13, 72). Acute cutaneous GvHD usually begins with erythematous, rashes on the ears, palms, and soles. Martin and coworkers reported results of 740 allogenic transplantations and 81% of patients with aGvHD had skin involvement (4). Damage to the skin could be defined by vacuolar degeneration of the basal cell layer, dyskeratotic keratinocytes, and mononuclear cell infiltrates (73). Epithelial damage occurs at the tips of rete ridges and hair follicles, regions where selective targeted apoptotic rete cells are located (74). A recent study by Paczesny et al. reported that elafin could be a potential biomarker for diagnosis and prognosis of skin GvHD (75).

Liver is another target organ of GvHD. Hepatic GvHD is manifested by abnormal liver function tests and a rise in the serum level of bilirubin and alkaline phosphatase. Donor lymphocytes attack the bile duct epithelial cells causing endothelialitis, pericholangitis, and apoptotic bile duct destruction (76). While liver GvHD affecting bile ducts and resulting in severe hyperbilirubinemia occurs less frequently, there is an increasing rate of hepatitis like cGvHD as another, but less harmful liver lesion (77).

Gastrointestinal tract represents the most severely affected organ after conditioning. GI GvHD is characterized by secretory and voluminous diarrhea, severe abdominal pain, vomiting, and anorexia (13). Snover and colleagues used immunohistochemistry to explain histologic features of the GI tract during GvHD (78). Single cell apoptosis was observed along with patchy ulcerations and apoptotic bodies in the base of crypts with loss of the surface epithelium (13, 78). The base of the intestinal crypts, where epithelial stem cells are located, is the most sensitive target for GvHD as it is the site of epithelium regeneration and Paneth cells. Recently, Levine and colleagues observed loss of the Paneth cells at the onset of GI GvHD (79) suggesting these cells as sensitive targets of GvHD. In addition, as stated earlier, it was proposed that regenerating islet-derived 3-a (reg3a), released from Paneth cells, was a potential plasma biomarker for lower GI GvHD (63), Paneth cell damage contributes to loss of antimicrobial peptides and accelerates the loss of microbial diversity in GvHD, a major risk factor of treatment-related mortality (80, 81).

## Further HSCT-Related Complications

### Overview

Although GvHD is the main complication of allogeneic SCT, non-relapse-related mortality (NRM) can occur independently from the occurrence of GvHD or in patients with minor GvHD. Overall, NRM has decreased in the last 10 years as a result of several improvements such as reduced intensity conditioning; resulting in reduced organ toxicity, improved donor selection and matching, and progress in supportive treatment (82).

Major complications include viral and fungal infections, which can occur independently from GvHD due to the immunodeficiencies induced by HSCT. GvHD and its treatment aggravate and prolong the risk of infectious complications, and many patients suffering from severe GvHD die from infectious complications. Beyond the period of acute GvHD, cGvHD and long-term complications are major causes of NRM and morbidity. Long-term complications include organ toxicities, endocrine deficiencies, and most important secondary cancers. HSCT patients’ survivors therefore need a long-term follow-up in order to allow early detection of complications, and several guidelines summarize the current recommendations (83, 84).

A detailed presentation of infectious complications, organ toxicities, and long-term complications is beyond the focus of this review; we therefore focus on the most relevant targets of complications: endothelial cells and pulmonary complications.

### Endothelial Complications

Endothelial complications occur clinically throughout the different phases of HSCT. In the early weeks after transplantation, sinusoidal obstruction syndrome (SOS) formally known as veno-occlusive disease (VOD) can result in severe liver damage and eventually multi-organ failure (85, 86). SOS results from conditioning-related toxicity in the sinusoids of the liver with subsequent occlusions by thrombosis and fibrosis. In the period of engraftment, cytokine storm-mediated capillary leakage syndrome can occur. With the introduction of calcineurin inhibitors (CNI) for prophylaxis of GvHD, which also give rise to some endothelial toxicity, transplant associated microangiopathy (TAM) has been increasingly observed during acute GvHD (87). Manifestations of intestinal TAM can mimic severe GvHD and provoke intestinal bleeding but require a different treatment regimen. Besides CNI-associated TAM, it can also occur as atypical hemolytic uremic syndrome, which results from a failure of cleaving von Willebrand Factor (88, 89). In long-term patients, cerebro- and cardiovascular complications are increased.

While clinical endothelial complications have been well known for many years, more recently the pathophysiology of endothelial cells in GvHD has been studied. *In vitro* models of endothelial cell cultures reveal that conditioning can induce endothelial apoptosis, which is aggravated by LPS-mediated inflammation and followed allogeneic cytotoxic T-cell damage (90). Murine models have demonstrated the role of endothelial neovascularization induced by conditioning leading to GvHD (91–93) and infiltrating donor T cells. Recently, Schmid et al. showed for the first time in a murine system that not only endothelial venules but also arterial vessels suffer direct endothelial damage during GvHD (94). Detailed studies in patients have shown an association of loss of dermal vessels, with CD8+ T cell infiltrates, demonstrating allogeneic reactions against endothelial cells (95, 96). More recently, endothelial damage has been shown to contribute to steroid resistance and failure to recover from GvHD. Loss of protective thrombomodulin was observed in biopsies from GvHD patients (97) together with increased serum thrombomodulin (98). In addition, genetic SNPs within the thrombomodulin gene have been identified as risk factors for GvHD (99). Finally, circulating endothelial factors such as angiopoietin levels pretransplant and VEGF levels posttransplant have been identified as risk factors of GvHD (100), which paves the way for infiltrating donor T cells.

### Pulmonary Complications

A further central target organ of HSCT-related complications is the lung. Early after transplantation, bacterial and fungal pneumonia are common, mainly due to *Aspergillus* predomination. In the posttransplant period of GvHD and immune reconstitution, viral pneumonia caused by CMV, respiratory viruses (influenza, parainfluenza, RSV, and metapneumovirus), and adenoviruses predominate as well as fungal pneumonia, especially in patients with severe immunosuppression (101). In addition, further infectious agents such as *Toxoplasma gondii* and *Pneumocystis jirovecii* causing toxoplasma and pneumocystis pneumonia, respectively, can cause pneumonia during the period of B cell reconstitution while B cell numbers are absent or low. Pneumonias caused by encapsulated bacteria such as pneumococci are also observed (102, 103). Early after HSCT, peri-engraftment respiratory distress syndrome causes rapid deterioration of respiratory functions during leukocyte recovery but responds rapidly to high dose corticosteroid treatment. In the initial stages of aGvHD, idiopathic pneumonia syndrome (IPS) is a serious complication resulting from conditioning-related toxicity and LPS-triggered allogeneic reactions. IPS may or may not be exacerbated by occult or unknown infections (89) and in either case, TNF blocking agents have been shown to be effective in both experimental models and in patients (86–88). The most frequent complication is bronchiolitis obliterans syndrome (BOS) characterized by inflammation of the small bronchiole with subsequent obstruction and lung destruction (104, 105). Early monitoring and intervention with topical corticosteroids, azithromycin and possibly systemic immunosuppression is needed to prevent progression to irreversible lung damage, which may lead a requirement for lung transplantation (106, 107). Besides BOS, restrictive changes can be observed such as pulmonary fibrosis, bronchiolitis obliterans organizing pneumonia, and pulmonary VOD (108).

Cellular therapy is one approach increasingly used as a second-line treatment. Tregs (109) and MSCs (110–112) are promising cellular products, but phase 3 trials are yet to be conducted.

## Recent Advances and Perspectives in GvHD

For over 30 years, immunosuppressive drugs have served as a central strategy to reduce GvHD. Drugs such as sirolimus, tacrolimus, and methotrexate are the mainstay in the treatment of GvHD (113). Complete *ex vivo* T-cell depletion is no longer routinely used in HLA-matched transplantation as it also largely abolishes GvL effects. A more recent report from Finke and colleagues suggested ATG as an *in vivo* T-cell depletion may be more efficacious in lowering the incidence of severe acute GvHD in matched and mismatched HSCT from unrelated donors while GvL effects seemed less affected (114). Hundred patients were enrolled in the study. Comparable outcomes were obtained for GvHD patients receiving bone marrow or peripheral blood stem cells from matched or one antigen mismatched-unrelated donors when ATG was added to the standard prophylaxis (cyclosporine + methotrexate) (114). The use of ATG may therefore contribute to balance GvH versus GvL effect and enable HLA mismatch donors to be used as well as fully match-unrelated donors, with no difference in outcome. As an alternative, elimination of alloreactive T cells by posttransplant cyclophosphamide may become an option, which is already widely used for GvHD prophylaxis following haploidentical transplantation (115). Whether this approach can be integrated in the HLA-identical setting as a potential alternative to calcineurin inhibitors is under current investigation.

Pathogen recognition receptors like NLRs and TLRs are known to control adaptive immune responses in inflammatory disorders (37), and the research on the role of these receptors has resulted in the description of the interaction of the microbiota and the immune system in the setting of GvHD. Loss of microbiome diversity early after HSCT has been recognized as a new risk factor for GvHD and HSCT-related complications (116). This observation suggests that restoration of a diverse microbiome could be a new approach to induce intestinal and systemic tolerance, and pre-/pro- and post-biotic strategies, as well as several approaches of fecal microbiota transplantation, that are currently being tested in both experimental and clinical settings of HSCT (41).

Regulatory T cells have been expanded *in vitro* and used for prophylaxis and treatment of GvHD in experimental and small clinical trials (117, 118). Another option is induction of Tregs in patients, e.g., by interleukin-2 (IL-2) (119). Induction of Tregs has also been postulated as one mechanism explaining the beneficial action of extracorporeal photopheresis for treatment of acute GvHD and cGvHD (120, 121). Besides Tregs, numerous alternative candidates for cellular therapy of GvHD exist such as MDSCs (122). MSCs are indirect immunoregulatory cells that induce tissue repair and show some promising activity in steroid-refractory GvHD (123, 124).

Among pharmacological agents, drugs with anti-inflammatory effects of corticosteroids but without numerous side effects are urgently needed. Recently, anti-inflammatory JAK2 inhibitors have shown promising effects both in GvHD and in rheumatology. Proteasome inhibitors and histone deacetylase inhibitors originally developed as anticancer drugs now show some promising activity in dampening T-cell responses (125). In cGvHD, the role of aberrant B cells is increasingly recognized, which paves the way for anti-B cell strategies like rituximab or new B cell development inhibitors like the Bruton’s tyrosine kinase-inhibitor ibrutinib (70).

A major issue in the treatment of aGvHD is that most approaches are initiated too late, when major changes have already severely damaged the target tissue. Therefore, biomarkers allowing early identification of patients at high risk are needed. A handful of biomarkers have been discovered, which might be used to guide treatment in the future (65).

Finally, the practice of stem cell transplantation differs between countries, within the same countries and between transplantation institutes. Approaches aimed at standardization of diagnosis and treatment are urgently needed, some of which have been addressed by several consensus projects (68, 126).

## Author Contributions

EH and AD designed and revised the review. SG provided the draft, summarized available data, selected the references, and wrote the review. DW, EM and XNW contributed to the review. All authors approved the final version of the manuscript.

## Conflict of Interest Statement

All authors declare that the research was conducted in the absence of any commercial or financial relationships that could be construed as a potential conflict of interest.
